# Utilizing *Chlorella vulgaris* algae as an eco-friendly coagulant for efficient removal of polyethylene microplastics from aquatic environments

**DOI:** 10.1016/j.heliyon.2023.e22338

**Published:** 2023-11-13

**Authors:** Afsaneh Esmaeili Nasrabadi, Mohaddeseh Eydi, Ziaeddin Bonyadi

**Affiliations:** a Student Research Committee, Department of Environmental Health Engineering, School of Health, Mashhad University of Medical Sciences, Mashhad, Iran; b Department of Environmental Health Engineering, School of Health, Mashhad University of Medical Sciences, Mashhad, Iran

**Keywords:** *Chlorella vulgaris*, Biocoagulation, Polyethylene, Microplastics

## Abstract

Polyethylene (PE) microplastics (MPs) are small particles of plastic made from polyethylene, which is a commonly used type of plastic. These microplastics can be found in water sources, such as rivers, lakes, and oceans. They are typically less than 5 mm in size. *Chlorella vulgaris* (*C. vulgaris*) is an excellent, simple and inexpensive biocoagulant that can effectively remove a wide range of pollutants through the coagulation and flocculation mechanism. In this study, *C. vulgaris* algae were used to remove PE MPs. The experiments were designed using the Behnken Box model. The evaluated parameters were the initial PE concentration (100–400 mg/L), the *C. vulgaris* dose (50–200), and the pH (4–10). The findings showed that increasing the concentration of polyethylene had a positive effect on the efficiency of removal. In addition, the dose of *C. vulgaris* and pH parameters were inversely and directly related to removal efficiency, respectively. The highest removal efficiency was observed under alkaline conditions. Overall, the maximum PE removal efficiency was 84 % when the concentration of PE was 250 mg/L, the dose of *C. vulgaris* was 50 mg/L, and the pH was 10. It can be concluded that algae can be used as an environmentally friendly coagulant for effectively removing MPs from aquatic environments.

## Graphical abstract

**Figure:**
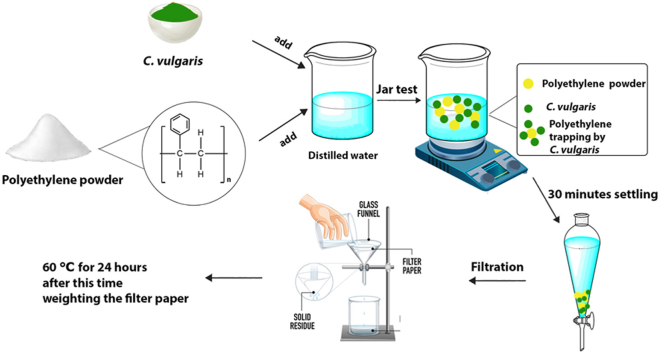

## 1 Introduction

The use of plastics is increasing day by day, as the production of these materials has significantly increased since the 1950s. In 2017, global plastic production reached 348 million tons [1]. Plastics in the environment break down over time and transform into small particles, known as MPs. Recently, water pollution has become a major concern for microplastics (MPs). These small plastic particles, ranging in size from 100 nm to 5 mm, enter the environment in two primary and secondary forms. Primary MPs are small particles that enter the environment as raw materials used in industry, while secondary MPs are produced from the mechanical or chemical degradation of plastic parts in the environment [2]. MPs enter a wide range of water bodies, including oceans, rivers, lakes, groundwater, sewage, and drinking water [3,4]. There are many types of MPs, but PE has the same density as natural water and is more commonly found in people's lives, posing potential threats [5]. The discovery of MPs in the aquatic environment reveals that almost all living organisms, including humans, are exposed to pollution from MPs [6,7]. The presence of MPs in marine species, resulting from the high consumption of seafood such as fish and shellfish, raises concerns about the potential effects of MPs on human health [8]. MPs can easily adsorb hazardous substances such as polycyclic aromatic hydrocarbon compounds (PAHs), cyanide [9], and pesticides [10], as well as rare earth metal elements, due to their large specific surface area and hydrophobic surface properties. This adsorption behavior of MPs can cause problems for human health, including endocrine disorders, liver damage, induction of oxidative stress, and changes in enzyme activities of different organisms with varying degrees of severity [11,12]. PE MPs are small particles of plastic made from PE, which is a commonly used type of plastic. These MPs can be found in water sources, such as rivers, lakes, and oceans [13]. The removal of MPs has been carried out using conventional methods, including chemical processes such as coagulation and photocatalysis. Zhang et al. (2021) removed MPs using active silicic acid and anionic polyacrylamide, achieving removal rates of 54.70 % and 91.45 %, respectively [14]. In another study, polystyrene (PS) and PE were removed through coagulation using polyaluminum chloride and FeCl3. The removal efficiency of PS and PE was 77.83 % and 29.70 %, respectively, at the dose of 90 mg/L polyaluminum chloride coagulant. With increasing the dose of this coagulant, the removal efficiency remained constant or decreased. In the case of FeCl3 coagulant, the highest removal efficiency for PS and PE was approximately 70 % and 20 %, respectively, which remained constant with the increase of coagulant dosage [15]. Rajala et al. (2020) successfully removed MPs using various chemical coagulants. The highest removal efficiency was 99.4 %. In addition, ferric chloride and polyaluminum chloride coagulants showed better performance than polyamine [16]. In general, it can be said that coagulation and flocculation processes in water treatment plants can remove 70–80 % of MPs from water [17]. However, despite their high removal efficiency, these methods come with significant operational and maintenance costs. Since it is crucial to maintain environmental balance, public health, and the food chain, there is a need to explore more efficient methods of removing MPs [18]. The use of natural coagulants, as opposed to chemical types, has been considered due to their compatibility with the environment [19]. In addition, the use of this type of coagulant can significantly reduce the amount of sludge generated during the water treatment process in comparison to mineral salts. In fact, the volume of sludge produced by natural coagulants is five times less than the volume of sludge produced by mineral salts. In addition, the sludge produced by natural coagulants is biodegradable and has high nutritional value. It can be used as a biofertilizer on land [20]. Microorganisms play a crucial role in the bioremediation process, which involves the removal of pollutants from water. Bioremediation is a process that utilizes natural microorganisms to degrade harmful substances into less toxic or non-toxic compounds [21]. Moreover, microorganisms can remove pollutants through processes such as biosorption, coagulation and flocculation, biofiltration, and biodegradation [22]. Algae-based natural methods have attracted attention for pollutant removal applications due to their renewable nature, low cost, availability, and high adsorption capacity [23,24]. Algae can effectively contribute to coagulation and flocculation processes due to the presence of functional groups, such as carboxyl, sulfonate, hydroxyl, and amine, on their cell walls [25,26].The compatibility of algae with the environment has made them a promising candidate for pollutant removal [27]. Microalgae are a diverse group of algae that are found in various aquatic environments. These microscopic organisms exist as single cells or small cell clusters in the environment. Microalgae remove various pollutants from water [28]. These microorganisms have the ability to absorb organic and mineral substances from water. In addition, they can attach themselves to the surface of suspended particles and form large flocs [29]. This process aids in the removal of different pollutants. In addition, the photosynthetic nature of microalgae enables them to colonize the interstitial spaces between sand particles [30]. This process of colonization acts as a natural filtration system, further purifying the water. Through photosynthesis, microalgae can utilize sunlight and carbon dioxide to generate oxygen, thereby improving the overall water quality [31]. *C. vulgaris* is a green microalga known for its high growth rate, resistance to changes in cultivation systems, and ease of production. These features make it an attractive option for removing various pollutants, including MPs, from aqueous solutions [32,33]. This green algae has been able to remove 95 % of arsenic, cadmium, cobalt, chromium, and iron from wastewater [34]. Sarmah et al. (2018) utilized two prevalent cyanobacterial species to degrade low-density polyethylene (LDPE) sheets. The results showed that 4 % of the carbon content of PE was decomposed by cyanobacteria [35]. Kumar et al. (2017) utilized microalgae, including green algae, blue-green algae, and diatoms, for the biodegradation of polyethylene. The highest percentage of degradation was attributed to blue-green algae, which decomposed 18.8 % of polyethylene [36]. Currently, no studies have been conducted on the use of *C. vulgaris* algae for the removal of PE MPs. We found that *C. vulgaris* had a significant effect on the removal of PE MPs by conducting smaller-scale experiments. This study investigates the bioremediation of PE using the algae species *C. vulgaris* as a comprehensive and environmentally friendly strategy.

## 2 Materials and methods

### 2.1 Chemicals and reagents

PE granules were purchased from Peshgaman Plastic Company, Iran. Sodium hydroxide and hydrochloric acid were obtained from Merck, Germany. *C. vulgaris* (ABDF 21144) was obtained from the National Algal Culture Collection of Iran. To prepare laboratory solutions with varying concentrations, double-distilled water was purchased.

### 2.2 Characteristics and measurements

Field emission scanning electron microscopy (FESEM) imaging was used to observe the changes in surface morphology of particles. The FESEM analysis was carried out using a Supra 55 electron microscope manufactured by Carl Zeiss in Germany. To determine the chemical composition, bonds, and functional groups of the MFs before and after sonication, Fourier transform infrared spectrometer (FTIR) analysis was performed using a PerkinElmer spectrometer, specifically the FT-IR/NIR FRONTIER model. Furthermore, energy-dispersive X-ray (EDX) analysis was employed to determine the elemental composition of the samples. This analysis was conducted using an Oxford device connected to a JEOL-JSM-5600 SEM.

### 2.3 *C. vulgaris* preparation

*C. vulgaris* cultivation was conducted in a 500 mL reactor filled with BG-11 medium, under a light intensity of 5000 lux and at room temperature (25 ± 2 °C) for a duration of 14 days. Then, the algal cells were separated from the culture medium by centrifugation at 5000 rpm for 10 min, dried at 60 °C for 24 h, and stored in a sterile and dark environment. This process ensures that the algae cells are free from contamination and can be used for further analysis or applications.

### 2.4 PE preparation

The PE granules were washed with double distilled water and 1 N HCl, dried at 60 °C for 24 h, crushed using a grinder, sized with a 425 μm sieve, and finally, stored in a dark environment to prevent direct contact with light and moisture.

### 2.5 Design of experiments

According to Table 1, various parameters were considered to evaluate the efficiency of PE removal by *C. vulgaris*. These parameters included the initial concentration of PE (100–400 mg/L), the dose of *C. vulgaris* (50–200 mg/L), and the pH level (4–10). The size of PE used in all experiments was less than 425 μm.

**Table 1:** Main parameters used in different values to remove PE.

Factor	Parameter level			
	Code	−1	0	+1
Microplastic (mg/L)	A	100	250	400
Algae (mg/L)	B	50	125	200
pH	C	4	7	10

To conduct the experiments, 200 cc of the reaction solution, which included predetermined quantities of PE and *C. vulgaris*, were combined using a jar machine. The machine was set to a speed of 400 rpm for 1 min to agitate the algae and PE, followed by a speed of 100 rpm for 15 min to allow clots to form. After completing these steps, the final solution is transferred to the Imhoff funnel and left undisturbed for 30 min to allow the biological clots containing MPs to settle. After the settling time, the supernatant was collected and filtered using a 0.45 μm Whatman filter. Finally, the filters were dried at 60 °C for 24 h. Equation (1) is used to calculate the amount of PE removal:

(1) $$R\;\left(\%\right)=\frac{M_{1}-M_{2}}{M_{1}}\times 100\%$$

Where M1 represents the initial weight of the PE before the removal process, and M2 represents the weight of the PE remaining on the filter paper after the removal process.

## 3 Results and discussion

### 3.1 Characterization

**Fig. 1:**
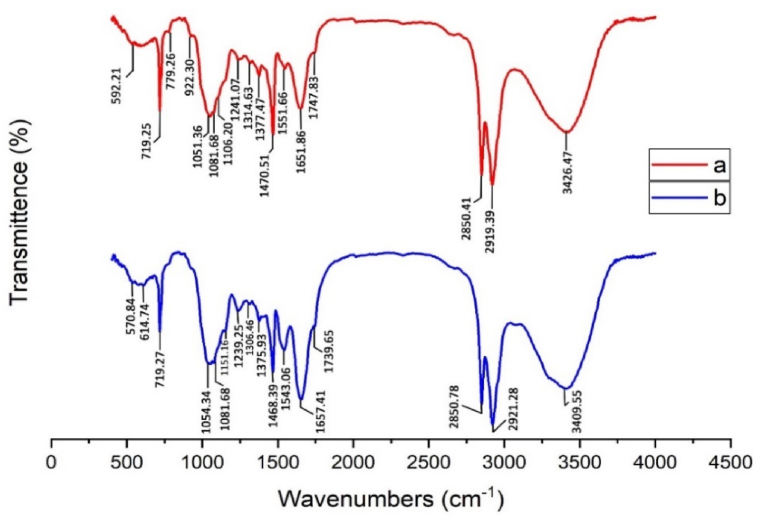
FTIR spectrum of (a) PE and (b) biological flocs.

**FT-IR:**Fig. 1 indicates FTIR spectrum of PE and biological flocs. As can be seen from Fig. 1, before PE removal, the peak at 3426.47 cm^−1^ is associated with the presence of O–H groups [15]. The peaks at 2919.39 cm-1 and 2850.41 cm-1 correspond to the asymmetric stretch of CH2 and the symmetric stretch of CH2, respectively [37]. The peak at 1747.83 cm^−1^ corresponds to the C=O stretching bands [38]. The peak at 1468 cm-1 represents the bending deformation, while the peak at 1373 cm-1 corresponds to the symmetric deformation of CH3 [37]. The peak observed at 719.29 cm-1 indicates the presence of the benzene ring, which has not changed significantly after removal [39]. According to Fig. 1, after the removal process, the peak corresponding to O–H groups decreased to 3409.55 cm^−1^. In this spectrum, the asymmetric stretch of CH_2_ has reached 2921.28 cm^−1^, indicating a significant increase compared to the peak observed before removal. The peak corresponding to the C=O group decreased from 1747.83 cm to 1 to 1739.65 cm-1. This decrease may be attributed to the formation of clots caused by the adsorption of PE onto the algae. The peak at 1657.41 cm-1 corresponds to the amine groups in *C. vulgaris*, which reduce the repulsive force between PE particles [40]. The peak at 1151.16 cm^−1^ is related to the bonding of C–*O*–C carbon groups and algal polysaccharides, indicating the formation of clots containing PE and *C. vulgaris* [40].

**Fig. 2:**
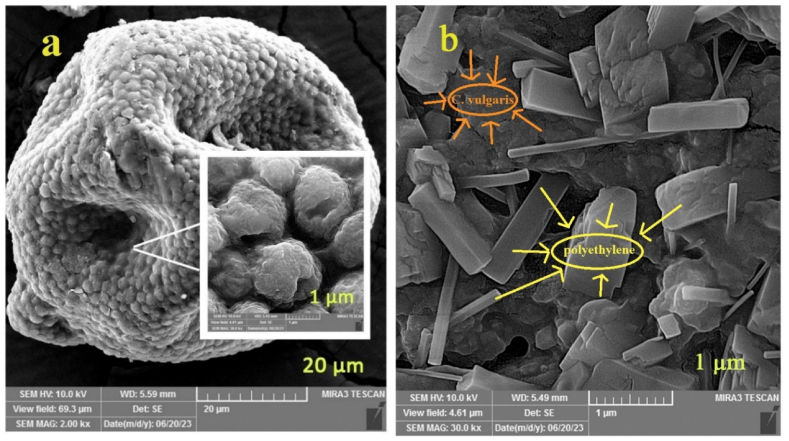
FESEM of (a) *C. vulgaris* and (b) biological flocs.

**FESEM:**Fig. 2a shows the FESEM image of *C. vulgaris* and biological folcs. From Fig. 2a, it can be observed that *C. vulgaris* appears as spherical particles with numerous pores and folds on its surface. These characteristics create a favorable environment for the absorption of pollutants, including MPs. Fig. 2b shows the FESEM image of the coagulation of PE particles after the process of coagulation by *C. vulgaris*. With a little precision, the relationship between MP particles and algae can be seen from the findings. In addition, PE particles accumulated in the pores and crevices of the algal surface, indicating the binding between MPs particles and algal cells [41]. This binding leads to the formation of large flocs, which ultimately increases the rate of floc settling.

**Fig. 3:**
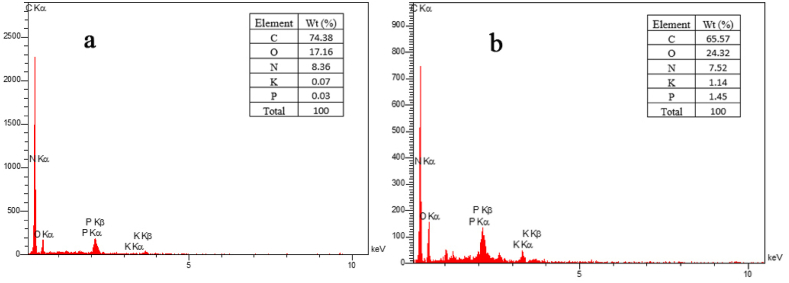
EDX spectrum of PE (a) before and (b) after removal.

**EDX:**Fig. 3 shows the percentage of the main constituents of PE before and after the removal process. According to Fig. 3a, the percentages of carbon, oxygen, nitrogen, potassium, and phosphorus elements were 74.38 %, 17.16 %, 8.36 %, 0.07 %, and 0.03 %, respectively. After the removal process, the values of the mentioned elements were changed. As shown in Fig. 3b, the carbon content decreased to approximately 65 %, while the oxygen content increased to around 24 %. Other elements experienced a change within a range of 1 % compared to before removal. These changes in the elements after the removal process can be attributed to the presence of *C. vulgaris*, which is attached to the PE particles [42,43].

**Zeta potential (ZP):** The zeta potential is a crucial factor in measuring the electrostatic dispersion and stability of particles. It helps determine the degree of repulsion or attraction between particles and predicts their behavior in various environments. When coagulant particles have a significantly positive or negative zeta potential (ζ > +30 mV or ζ < −30 mV), they repel each other, which results in dispersion stability. On the other hand, when the zeta potential is minimal (−30 mV < ζ < +30 mV), there may be no significant force preventing particle aggregation, which leads to the instability of the dispersion. It is therefore essential to understand the zeta potential of particles in order to predict their behavior and design effective strategies for their removal from aqueous solutions. The values of zeta potential for PE particles and biological flocs are presented in Table 2. The results show that the zeta potential values for PE and biological flocs are −35.6 and −19.3, respectively. These values indicate that the repulsive force is significantly reduced after the removal process. In other words, there is a possibility of particle accumulation and the formation of larger clusters that can remove them from the water.

**Table 2:** Zeta potential and DLS values for PE, and PE flocs.

Material	Zeta potential (mV)	Average diameter (μm)
PE	−35.6	0.70
PE floc	−19.3	5.16

**DLS:** According to the findings in Tables 2 and it is shown that the average diameter of PE particles before the removal process is 0.70 μm. After the removal process, the average diameter of the formed flocs increases significantly to 5.16 μm. These results confirm that PE particles were removed from the environment through the coagulation and flocculation mechanisms [44]. The findings show that using *C. vulgaris* as a biocoagulant can be a promising method for removing PE particles from aqueous solutions.

### 3.2 Response model

In the present study, the primary objective was to investigate the effect of *C. vulgaris* on the removal of PE from aqueous solutions. The outcomes are documented in Table 3, which demonstrates the effectiveness of *C. vulgaris* in removing PE.

**Table 3:** BBD matrix for PE removal by *C. vulgaris*.

Run No	Coded variable			Removal (%)	Run No	Coded variable			Removal (%)
	A	B	C			A	B	C	
1	0	0	0	53.8	10	0	0	0	50.8
2	1	−1	0	47.37	11	0	0	0	50.2
3	0	0	0	55	12	−1	1	0	18
4	0	−1	−1	40.2	13	0	1	−1	45.8
5	0	0	0	46.2	14	−1	−1	0	53.5
6	−1	0	−1	23.5	15	1	1	0	40.12
7	1	0	−1	38.1	16	1	0	1	60.37
**8**	0	−1	1	84	17	−1	0	1	57.5
9	0	1	1	55.6					

Based on the results of Table 3, the maximum and minimum efficiency of PE removal were 84 % and 18 %, respectively. Table 4 shows the evaluation of statistical models for adequacy, including linear, 2FI, quadratic, and cubic models. A quadratic model was proposed to fit the data from Table 4.

**Table 4:** Statistical adequacy evaluation of models.

Source	Sequential p-value	Lack of Fit p-value	Adjusted R^2^	Predicted R^2^
Linear	0.0037	0.0159	0.5488	0.2580
2FI	0.1342	0.0224	0.6558	0.0007
Quadratic	0.0019	0.3575	0.9349	0.7425
Cubic	0.3575		0.9451	

Table 5 displays the coefficients of the quadratic model for PE removal by *C. vulgaris.*

**Table 5:** Coefficients of estimation for quadratic model of PE removal by *C. vulgaris*.

Factor	Coefficient Estimate	df	Standard Error	95 % CI Low	95 % CI High	VIF
Intercept	51.20	1	1.68	47.24	55.16	
A- MP Conc.	4.18	1	1.32	1.05	7.31	1
B– *C. vulgaris* Dose	−8.19	1	1.32	−11.33	−5.06	1
C- pH	13.73	1	1.32	10.60	16.87	1
AB	7.06	1	1.87	2.63	11.49	1
AC	−2.93	1	1.87	−7.36	1.50	1
BC	−8.50	1	1.87	−12.93	−4.07	1
A^2^	−11.49	1	1.83	−15.81	−7.17	1.01
B^2^	0.0400	1	1.83	−4.28	4.36	1.01
C^2^	5.16	1	1.83	0.8422	9.48	1.01

From Table 5, the coefficients obtained for each of the coded factors have been used to represent the quadratic model of PE removal efficiency (Y %). This model is expressed by the following formula:

(3) Y % = 51.20 + 4.18A–8.19B + 13.73C + 7.06AB – 2.93AC – 8.50BCE – 11.49A^2^ + 0.0400B^2^ +5.16C^2^

As per relation 3, each model consists of two parts - fixed and variable. Hence, the reduction of PE was measured at 51.20 %, which was influenced by several factors. The coded variables A, B, and C had coefficients of +4.18, −8.19, +12.60, and +13.73, respectively. The pH values, indicated by the C code and a coefficient of +13.73, had the greatest influence on PE removal. BC had the highest interaction coefficient of −8.50, while the parameter with the highest square influence was A^2^, with a value of −11.49. Table 6 shows the ANOVA values for the quadratic model of the response surface.

**Table 6:** Analysis of variance (ANOVA) for quadratic model of PE removal by *C. vulgaris*.

	Sum of Squares	df	Mean Square	F-value	p-value
**Model**	3353.02	9	372.56	26.54	0.0001
A- MP Conc.	139.95	1	139.95	9.97	0.0160
B– *C. vulgaris* Dose	537.10	1	537.10	38.26	0.0005
C- pH	1508.93	1	1508.93	107.48	<0.0001
AB	199.52	1	199.52	14.21	0.0070
AC	34.40	1	34.40	2.45	0.1615
BC	289.00	1	289.00	20.59	0.0027
A^2^	556.12	1	556.12	39.61	0.0004
B^2^	0.0067	1	0.0067	0.0005	0.9831
C^2^	112.11	1	112.11	7.99	0.0256
Residual	98.28	7	14.04		
Lack of Fit	50.92	3	16.97	1.43	0.3575
Pure Error	47.36	4	11.84		
Cor Total	3451.30	16			
R^2^	0.97		Predicted R^2^	0.74	
Adjusted R^2^	0.93		Adeq Precision	23.14	

**Fig. 4:**
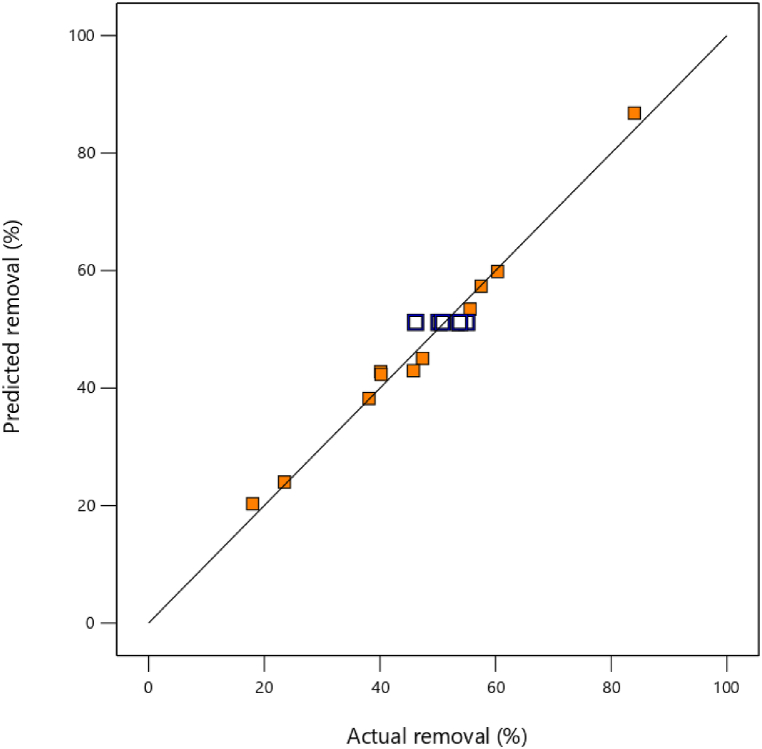
Graph of predicted removal against actual removal.

The findings of Table 6 indicated that the model was significant (*P*-value <0.05). The R^2^, adjusted R^2^, predicted R^2^, and adequacy precision values were 0.97, 0.93, 0.74, and 23.14, respectively. The adequacy precision term calculates the signal-to-noise ratio. This factor was 23.14, which is greater than the minimum desired value of 4. Fig. 4 shows the graph of predicted removal against actual removal. As indicated from Fig. 4, it is clear that the model is adequate in providing a good prediction for PE removal.

### 3.3 Effect of main factors on removal efficiency

**Fig. 5:**
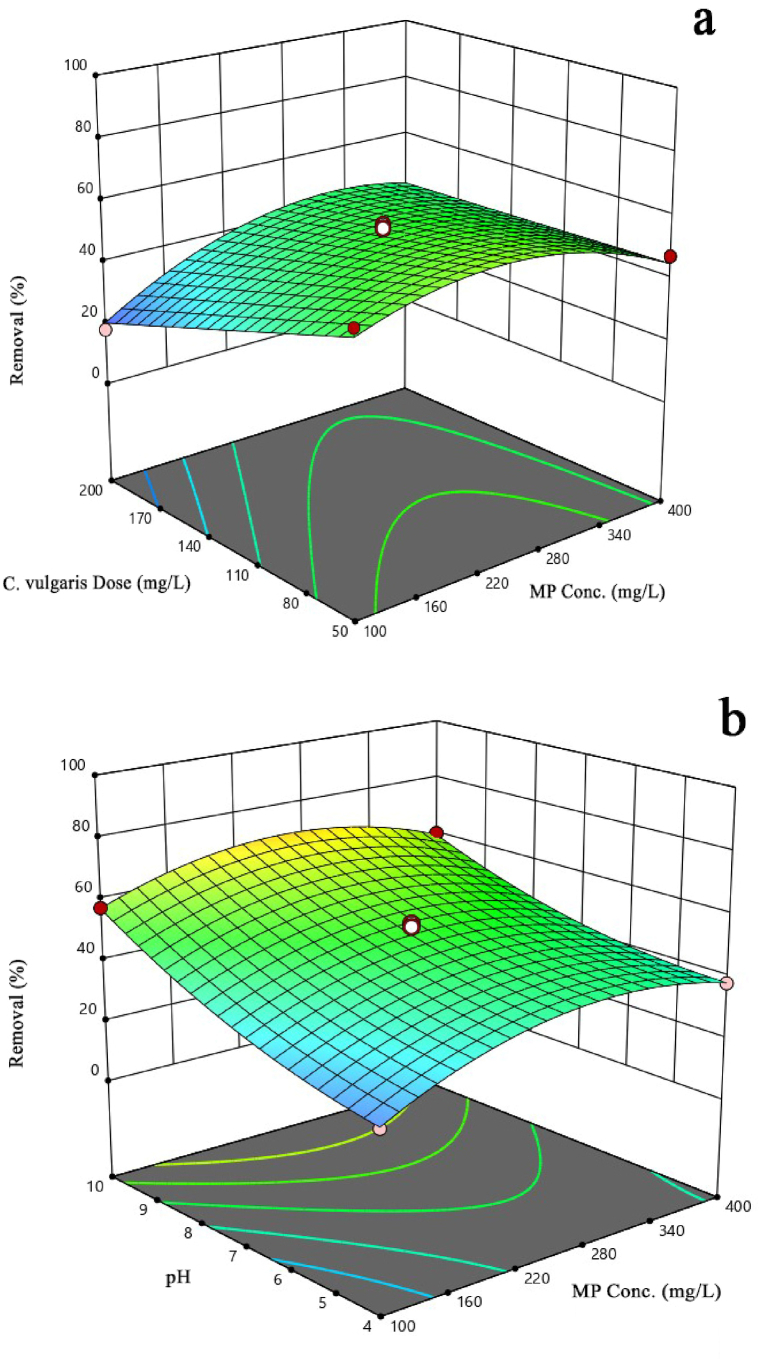
The 3D Response surface plot about the effects of (a) *C. vulgaris* dose vs. PE concentration and (b) pH vs. PE concentration.

Fig. 5 shows the effect of the initial PE concentration, *C. vulgaris* dose, and pH on the rate of PE removal. A point to note is that when interpreting the effect of one parameter on the response, it is assumed that the other parameters are zero.

### 3.4 Effect of *C. vulgaris* dose

The effect of *C. vulgaris* in doses ranging from 50 to 200 mg on PE removal was evaluated. The results of Fig. 5a show an inverse relationship between the dose of *C. vulgaris* and the rate of PE removal. Thus, by increasing the coagulant dose to 200 mg/liter, the removal efficiency reaches its lowest level, which is 18 %. On the other hand, when the algae are exposed to a dose of 50 mg/liter, the removal efficiency increases to 84 %. Low doses of *C. vulgaris* result in the formation of smaller clots in association with PE. At low doses of *C. vulgaris*, the algal cells sporadically come into contact with PE particles, leading to the formation of small, lightweight flocs. This, in turn, results in poor sedimentation. Due to this fact, it can be concluded that there is a greater surface area available for the absorption of MPs, leading to an increase in removal efficiency [45]. In one study, it was discovered that increasing the concentration of the coagulant leads to charge reversal and restabilization of the particles [46].

### 3.5 Effect of initial PS concentration

PE concentration is another factor that affects PE. Fig. 5a shows that the removal efficiency increased as the concentration of PE increased from 100 mg/L to 250 mg/L (p-value <0.05). This can be explained by the fact that at high concentrations of PE, the likelihood of contact between PE particles and *C. vulgaris* increases. As a result, large clots form and settle, leading to the removal of PE [47]. But as the concentration of PE increases from 250 to 400 mg/L, the removal efficiency decreases. This shows that within the optimal PE concentration range, the efficiency of PE removal is maximized. However, beyond this range, the removal efficiency decreases. At high concentrations of PE, these MPs can form a protective layer around themselves, reducing contact between the MPs and algal cells. Moreover, at high concentrations of MPs, the repulsive force between the particles contributes to their stability and makes their removal challenging [17].

### 3.6 pH effect

The pH of the solution plays an important role in determining the stability of the formed flocs [45]. As shown in Fig. 5b, increasing the pH has a positive effect on the removal efficiency. The highest removal efficiency (84 %) was obtained at pH 10. The findings of Tang et al. (2023) indicated that the removal efficiency of MPs decreased to less than 30 % at pH levels below 7, while it increased by 51.33 % at pH levels above 7 [48]. A possible explanation for this phenomenon is the influence of pH on the surface charge of MPs. In an alkaline pH environment, the surface charge of MPs becomes more negative, which can impact their ability to adsorb onto algae that have a positive surface charge [49]. At pH 10, OH- ions are present in the solution and they react with surface agents of algae, resulting in neutralization. This process can lead to the formation of significant biological flocs, which eventually settle [50].

### 3.7 Process optimization

To achieve optimal efficiency in PE removal, the Behnken's Box method was used to evaluate the data. Based on this, a maximum efficiency of 84 % was obtained at a PE concentration of 250 mg/L, a *C. vulgaris* dose of 50 mg/L, and a pH of 10.

## 4 Conclusion

In the present study, *C. vulgaris* algae were used as a biological coagulant to remove polyethylene. The parameters of the initial PE concentration, *C. vulgaris* dose, and pH were evaluated to determine the optimal conditions for removal. The experiments were designed using Design Expert software and the Behnken Box model. The results showed that increasing the concentration of PE had a positive effect on the efficiency of removal. In addition, the dose of *C. vulgaris* and pH parameters were inversely and directly related to removal efficiency, respectively. The highest removal efficiency was observed under alkaline conditions. Overall, the maximum PE removal efficiency was 84 % when the concentration of PE was 250 mg/L, the dose of *C. vulgaris* was 50 mg/L, and the pH was 10. It can be concluded that algae can be used as an environmentally friendly coagulant for effectively removing MPs from aquatic environments.

## Funding statement

This work was supported by 10.13039/501100004748Mashhad University of Medical Science (Iran) [4001511].

## Data availability statement

Data will be made available on request.

## CRediT authorship contribution statement

**Afsaneh Esmaeili Nasrabadi:** Writing – original draft. **Mohaddeseh Eydi:** Methodology. **Ziaeddin Bonyadi:** Writing – review & editing, Supervision, Methodology, Conceptualization.

## Declaration of competing interest

The authors declare no conflict of interest.
